# Influence of temperature, mixing, and addition of microcystin‐LR on microcystin gene expression in *Microcystis aeruginosa*

**DOI:** 10.1002/mbo3.393

**Published:** 2016-07-14

**Authors:** Pia I. Scherer, Uta Raeder, Juergen Geist, Katrin Zwirglmaier

**Affiliations:** ^1^ Aquatic Systems Biology UnitLimnological Research Station IffeldorfDepartment of Ecology and Ecosystem ManagementTechnical University of MunichMunichGermany; ^2^ Present address: Bundeswehr Institute of MicrobiologyMunichGermany

**Keywords:** gene expression, *mcyB*, *mcyD*, *Microcystis aeruginosa*, qPCR, toxic cyanobacteria

## Abstract

Cyanobacteria, such as the toxin producer *Microcystis aeruginosa*, are predicted to be favored by global warming both directly, through elevated water temperatures, and indirectly, through factors such as prolonged stratification of waterbodies. *M. aeruginosa* is able to produce the hepatotoxin microcystin, which causes great concern in freshwater management worldwide. However, little is known about the expression of microcystin synthesis genes in response to climate change‐related factors. In this study, a new RT‐qPCR assay employing four reference genes (*GAPDH*,* gltA, rpoC1,* and *rpoD*) was developed to assess the expression of two target genes (the microcystin synthesis genes *mcyB* and *mcyD*). This assay was used to investigate changes in *mcyB* and *mcyD* expression in response to selected environmental factors associated with global warming. A 10°C rise in temperature significantly increased *mcyB* expression, but not *mcyD* expression. Neither mixing nor the addition of microcystin‐LR (10 μg L^−1^ or 60 μg L^−1^) significantly altered *mcyB* and *mcyD* expression. The expression levels of *mcyB* and *mcyD* were correlated but not identical.

## 1 Introduction

*Microcystis aeruginosa* is a cyanobacterium found in waterbodies worldwide (Chorus & Bartram, 1999; Moreira, Spillane, Fathalli, Vasconcelos, & Antunes, 2014; van Gremberghe et al., 2011). Many strains of *M. aeruginosa* produce the toxin microcystin, which is harmful to humans, livestock, and aquatic animals. Microcystin is actively taken up by hepatocytes and is, therefore, classified as a liver toxin (Runnegar & Falconer, 1982). Toxic *Microcystis* blooms have been associated with human and livestock fatalities or disease (Carmichael et al., 2001; Van Halderen et al., 1995), and microcystin‐producing cyanobacterial blooms regularly pose a threat to the safety of the water supply (Hudnell, 2010).

Toxic cyanobacterial blooms are expected to occur more often in the future because of global warming (Paerl & Huisman, 2008, 2009; Paerl & Paul, 2012). Direct effects, such as rising temperatures, and indirect effects, such as intensified stratification, favor cyanobacterial blooms, that might be toxic in some cases. Increased water temperature is expected to give cyanobacteria a selective advantage over competing phytoplankton because of the high optimal growth temperature of studied cyanobacterial species (Elliott, Jones, & Thackeray, 2006; Jöhnk et al., 2008). Also, some findings suggest that toxic *Microcystis* genotypes are favored over nontoxic ones at warmer temperatures (Davis, Berry, Boyer, & Gobler, 2009). However, the link between temperature and toxin production remains largely unknown (El‐Shehawy, Gorokhova, Fernández‐Piñas, & del Campo, 2012). Consequently, increased temperature is one of the stressors related to climate change that we chose to investigate as a possible trigger of increased *M. aeruginosa* toxigenicity. Another change expected to be brought about by global warming is a shift in waterbody perturbation patterns. Model‐based analyses forecast increased water column stability and a warmer epilimnion (Wahl & Peeters, 2014), as well as an earlier seasonal stratification of waterbodies (Peeters, Straile, Lorke, & Livingstone, 2007). These effects will be caused, in part, by a shorter annual duration of ice cover (Magnuson et al., 2000). These developments are expected to favor cyanobacterial blooms (Jöhnk et al., 2008) because many cyanobacteria, such as *Microcystis*, are able to thrive under those conditions by actively regulating buoyancy and finding their optimal depth in the water column (Huisman et al., 2004; Walsby, Hayes, Boje, & Stal, 1997). On the contrary, intensified vertical mixing of the epilimnion caused by more frequent wind stress and extreme weather events, such as storms and floods, is expected for temperate regions (Gastineau & Soden, 2009; Helbling, Banaszak, & Villafane, 2015). Because the effect of such profound environmental changes on cyanobacterial toxicity or toxigenicity is unknown, we selected the process of mixing as another environmental stressor to investigate with regard to *M. aeruginosa* toxigenicity. Other factors, such as rising carbon dioxide levels (Wannicke et al., 2012) and increased eutrophication (Huisman, Matthijs, & Visser, 2005) of waterbodies, have also been linked to the enhanced growth of cyanobacteria. Taken together, these factors might not only enhance the growth of toxic cyanobacteria but also increase toxin concentrations in the environment. Thus, we investigated microcystin at different concentrations as a third environmental stressor that is expected to influence *M. aeruginosa* toxigenicity.

Microcystin, a toxin commonly associated with toxic cyanobacterial blooms, is a cyclic heptapeptide that is produced nonribosomally at a multienzyme complex. The genes necessary for the synthesis of microcystin are organized in a gene cluster with two bidirectionally transcribed operons (*mcyA‐C* and *mcyD‐J*) (Neilan et al., 1999; Tillett et al., 2000). Not all strains of *M. aeruginosa* have the genetic capability to produce microcystin. Unfortunately, toxic and nontoxic genotypes cannot be distinguished by means of microscopy (Komárek, 1991). Based on the knowledge about the genetic basis of microcystin synthesis, PCR‐based assays have been developed to distinguish toxic from nontoxic strains at the molecular level (Al‐Tebrineh et al., 2012; Fortin et al., 2010; Hautala et al., 2013; Hisbergues, Christiansen, Rouhiainen, Sivonen, & Borner, 2003; Kurmayer & Kutzenberger, 2003; Ostermaier & Kurmayer, 2010; Pimentel & Giani, 2014; Rantala et al., 2006). The detection and quantification of toxicity genes are of special interest because the direct detection of microcystin has certain limitations and drawbacks. More than 90 isoforms of microcystin, which differ in their two L‐amino acids or the presence of methyl groups on D‐erythro‐β‐methylaspartic acid (D‐MeAsp) and N‐methyldehydroalanine (Mdha), are known (Neilan et al., 2008; Welker & von Döhren, 2006). Commonly used enzyme‐linked immunosorbent assays (ELISAs) usually measure only one of the microcystin isoforms (microcystin‐LR) and have a certain degree of cross reactivity with other isoforms (Kaushik & Balasubramanian, 2013; Sangolkar, Maske, & Chakrabarti, 2006). Other methods, such as high pressure liquid chromatography (HPLC) or liquid chromatography–mass spectrometry (LC–MS), can distinguish several isoforms, but are also more expensive and labor‐intensive. Another limitation of these methods is the fact that methanol extracts are usually measured; therefore, protein‐bound microcystin is not detected (Meissner, Fastner, & Dittmann, 2013).

The genes encoding the proteins required to synthesize the secondary metabolite microcystin are not constitutively expressed (Wood, Rueckert, Hamilton, Cary, & Dietrich, 2011). Therefore, expression of the genes belonging to the microcystin gene cluster has been of interest for some time. Several approaches have been employed to study gene expression in *M. aeruginosa* in general and the expression of toxicity genes in particular. Microarrays, for example, have led to a deeper understanding of the systemic effects of microcystin on cells (Makower et al., 2015). Methods such as the RNase protection assay (Kaebernick, Neilan, Börner, & Dittmann, 2000) or competitive reverse‐transcriptase polymerase chain reaction (RT‐PCR) (Kim, Kim, Ahn, Yoo, & Lee, 2005) have been used to quantify *mcyB* and *mcyD* transcripts in the past. Reverse‐transcriptase quantitative polymerase chain reaction (RT‐qPCR) has been used to understand the impact of nutritional and other factors on the expression of microcystin genes. For instance, the influence of micro‐ and macronutrients on *mcyD* expression was investigated (Kuniyoshi, Sevilla, Bes, Fillat, & Peleato, 2013; Pimentel & Giani, 2014; Sevilla et al., 2008, 2010), and the effect of high light stress as well as co‐occurring cyanobacteria and cell concentration on *mcyE* expression was studied (Ngwa, Madramootoo, & Jabaji, 2014; Tran, Bernard, Ammar, Chaouch, & Comte, 2013; Wood et al., 2011).

Most of the previous RT‐qPCR studies, while making valuable contributions to our understanding of the microcystin synthesis gene expression dynamics of *M. aeruginosa*, normalized gene expression to a single reference gene; this single reference gene was mostly the 16S rRNA gene (Kuniyoshi et al., 2013; Pimentel & Giani, 2014; Sevilla et al., 2008). Although it is possible to use a single stably expressed gene as a reference, some scientists have found that the 16S rRNA gene is not a suitable reference gene (Radonić et al., 2004; Tran et al., 2013). Other studies have relied on a panel of several reference genes to average out small fluctuations in reference gene expression (Tran et al., 2013; Zhao et al., 2011). To the best of our knowledge, there is no RT‐qPCR assay for the microcystin synthesis genes of *M. aeruginosa* that employs a whole reference gene panel to assess the expression of one or more target genes. This led us to develop a novel RT‐qPCR assay with a panel of four reference genes (*GAPDH*,* gltA, rpoC1,* and *rpoD*) that can be used to assess the expression of two microcystin synthesis genes (*mcyB* and *mcyD*).

In summary, the aims of this study were (1) to design laboratory experiments suitable to test the hypothesis that those stressors increase microcystin gene expression and (2) to determine the fold change in *mcyB* and *mcyD* gene expression in response to elevated temperature, mixing regimes, and treatment with microcystin‐LR.

## 2 Material and Methods

### 2.1 Experimental design

Cyanobacterial cultures were grown to early exponential phase under laboratory conditions and then divided into two cohorts. One cohort continued growth under the original conditions and acted as a control, whereas the other was treated with a stressor. Three different stressors were tested: temperature rise, mixing, and the addition of microcystin‐LR. To investigate the effects of elevated temperature, cultures grown at 20°C were shifted to 30°C. To investigate the effect of mixing, liquid cultures were stirred in such a way that a vortex in the liquid was just about not forming (4 cm stir bar, 150 rpm). This mimics, to a degree, the perturbation of cyanobacteria in waterbodies subjected to mixing events such as storms or floods. To test whether increased levels of extracellular microcystin‐LR have an effect, two different concentrations of microcystin‐LR were added to batch cultures. Both concentrations tested (10 μg L^−1^ and 60 μg L^−1^) are well above the safe level (1 μg L^−1^) for drinking water (WHO, 2011) and within a range that can be realistically found in surface waters (Backer et al., 2008, 2010; Lee, Rollwagen‐Bollens, Bollens, & Faber‐Hammond, 2015). Three independent biological replicates were analyzed for each experimental condition. RNA was extracted from cultures that were harvested 72 hr after stressor application. Expression of the target genes *mcyB* and *mcyD* was assessed using RT‐qPCR with two technical replicates. Each experiment was repeated a minimum of two times.

### 2.2 Strains and Cultivation Techniques

*M. aeruginosa* strain SAG14.85, which is known to produce microcystin (Lyra et al., 2001), was obtained from the Culture Collection of Algae at Göttingen University in Germany (SAG). *M. aeruginosa* cells were grown in batch cultures in 300 ml Erlenmeyer flasks made of borosilicate glass. Cultures were grown in 200‐ml volumes of BG‐11 medium (Rippka, Deruelles, Waterbury, Herdman, & Stanier, 1979) supplemented with 0.5 mmol L^−1^ ammonium chloride under a light–dark regime of 14 hr light and 10 hr dark. MASTER TL5 HO 39W/865 1SL fluorescent light tubes emitting cool daylight (Phillips, Amsterdam, Netherlands) were used as light sources. The light intensity was 130 μmol s^−1 ^m^−2^ for both control and treated cultures (measured with a PAR sensor, LI‐COR, Lincoln, USA). Cultivation temperatures of 20°C and 30°C were used during the temperature rise experiments. The intermediate temperature of 25°C was used during the mixing and microcystin addition experiments for both control and treated cultures. Cyanobacterial growth was monitored by measuring the optical density at 730 nm (OD_730_) using a spectrophotometer (model 150‐20; HITACHI, Chiyoda, Japan), and cultures were regularly checked for contamination under a microscope (LEICA DM R; Leica Microsystems, Wetzlar, Germany).

### 2.3 Harvesting and RNA extraction

Harvesting was performed as follows: Twenty milliliter culture samples were filtered through a 0.2‐μm pore‐size cellulose nitrate filter (Sartorius, Göttingen, Germany), and then the filters, which retained the cyanobacteria, were frozen immediately at −80°C. Storage at −80°C did not exceed 14 days.

RNA extraction was performed as described by Penn, Wang, Fernando, and Thompson (2014), with minor modifications. In short, filters were cut, placed into 1.5 ml reaction tubes, and vortexed with 1 ml lysozyme solution (15 mg ml^−1^). After incubation for 10 min at 37°C, 2 g of 1.8–2.0‐mm ceramic beads (Sigmund Linder, Warmensteinach, Germany) were added, and the mixture was beaten for 10 min using a Mikro‐dismembrator II (Braun, Melsungen, Germany), submerging the tubes in ice every 2 min for 30 s. The resulting liquid was transferred to a fresh tube and subjected to centrifugation at 6,200*g* at 4°C for 3 min. The pellet was resuspended in 1 ml TRIsure (Bioline, Luckenwalde, Germany) and incubated for 5 min at 20°C. Addition of 0.2 ml chloroform and mixing was followed by 8 min of incubation and centrifugation at 13,800 *g* for 15 min. The upper phase was transferred into a fresh tube, and the chloroform wash was repeated. Addition of 1 μl of 15 μg/μl glycogen and 0.5 ml of isopropanol was followed by overnight incubation at −80°C. Subsequently, the samples were defrosted, mixed, and centrifuged at 18,800*g* for 30 min. The resulting pellet was washed with ethanol, dried, and resuspended in DEPC‐treated water.

The isolated RNA was cleaned up with the RNA Clean & Concentrator kit (Zymo Research, Irvine, USA) and stored at −80°C. Total RNA was quantified and checked for purity using a NanoVue Plus spectrophotometer (GE healthcare, Little Chalfont, UK) (Table S1), and its integrity was checked on a 1% agarose gel stained with GelRed (Biotium, Hayward, USA).

### 2.4 Primer design and testing

Several possible reference genes were evaluated in this study to assemble a suitable reference gene panel. Criteria for selecting reference gene primer pairs are listed in Table S2. These potential reference genes included the 16S rRNA gene and the genes encoding glyceraldehyde‐3‐phosphate dehydrogenase (*GAPDH*)*,* citrate synthase (*gltA*), DNA gyrase subunit B (*gyrB*), DNA‐directed RNA polymerase subunit gamma (*rpoC1*), and RNA polymerase sigma factor RpoD (*rpoD*). Primers targeting *GAPDH, gltA*,* gyrB,* and *rpoD* of *M. aeruginosa* NIES843 (Gen Bank AP009552.1) were designed using Primer3 software (Rozen & Skaletsky, 2000). Both primers designed in this study and primers obtained from the literature (Table 1) were checked for specificity using the BLAST algorithm (Altschul, Gish, Miller, Myers, & Lipman, 1990), and no homology to unwanted targets was found. Melting curves and agarose gel electrophoresis were used to ensure the specificity of the reaction under the chosen conditions (see Figs. S1 and S2). The efficiency of qPCR reactions and the *R* ^*2*^ values of standard curves were determined for all primers included in the assay (see Table S3 and Fig. S3). Oligonucleotides were synthesized by biomers.net (Ulm, Germany).

**Table 1:** Information about genes and primers in this study

Target gene	Primer name	Sequence 5′ to 3′	Source
*mcyB*peptide synthetase	mcyB30F	CCTACCGAGCGCTTGGG	(Kurmayer & Kutzenberger, 2003)
	mcyB108R	GAAAATCCCCTAAAGATTCCTGAGT	
*mcyD*polyketide synthase	RmcyDF	ACCCGGAACGGTCATAAATTGG	(Sevilla et al., 2008)
	RmcyDR	CGGCTAATCTCTCCAAAACATTGC	
*16S‐rRNA*16S ribosomal RNA	16S‐For	TGCGTAGAGATTGGGAAGAACATC	(Sevilla et al., 2008)
	16S‐Rev	GCTTTCGTCCCTGAGTGTCA	
*GAPDH*glyceraldehyde‐3‐phosphate dehydrogenase	GAPDH727F	GTTTCGGCGGTGGATTTAACC	This study
	GAPDH825R	ACCTTTCATCGGACCTTCG	
*gltA*citrate synthase	gltA429F	AGGTAATCATCCCATTCAGCCC	This study
	gltA528R	AACTTTCGCCGCTAAATCCG	
*gyrB*DNA gyrase subunit B	gyrB1041F	AGTCCGGGGTATTGTTGATTCC	This study
	gyrB1124R	ATAATCGTGTCGGCTACTTGGG	
*rpoC1*DNA‐directed RNA polymerase subunit gamma	rpoC1F	CCTCAGCGAAGATCAATGGT	(Alexova et al., 2011)
	rpoC1R	CCGTTTTTGCCCCTTACTTT	
*rpoD*RNA polymerase sigma factor RpoD	rpoD230F	GCAGGATTCGGTTATTGAGAGC	This study
	rpoD354R	CTGTTTTCCCCATTCAGCATCG	

The suitability of a gene to serve as reference gene under a certain experimental condition was verified using geNorm software (Vandesompele et al., 2002).

### 2.5 RT‐qPCR

Prior to reverse transcription, the RNA samples were freed from residual genomic DNA using DNase I digestion according to the manufacturer's instructions (Thermo Fisher, Waltham, USA).

Reverse transcription was performed using the High‐Capacity cDNA Reverse Transcription Kit (Applied Biosystems, Waltham, USA) according to the manufacturer's instructions. A no‐reverse‐transcriptase control reaction, which acted as a negative control for reverse transcription, was performed for every sample by omitting reverse transcriptase from the reaction. cDNA was stored at −20°C until analysis commenced.

The qPCR analyses were performed using a BioRad CFX 96 cycler, SsoAdvanced universal SYBR Green supermix (BioRad, Hercules, USA) and primers at 0.2 μmol L^−1^ in a 20‐μl reaction. Primer pairs for the target genes *mcyD* and *mcyB* were RmcyDF/RmcyDR (Sevilla et al., 2008) and mcyB30F/mcyB108R (Kurmayer & Kutzenberger, 2003), respectively. Primer pairs for the reference genes *rpoC*,* gltA*,* rpoD,* and *GADPH* were rpoC1F/rpoC1R (Alexova, Haynes, Ferrari, & Neilan, 2011), gltA429F/gltA528R, rpoD230F/rpoD354R, and GAPDH727F/GAPDH825R, respectively (Table 1). A 1‐μl volume of cDNA (50 ng) was used as the template. The cycling conditions were as follows: 95°C for 10 min, 40 cycles of 95°C for 15 s and 64.7°C for 1 min, and finally, a melt curve of 95°C for 10 min, and then a ramp from 65°C to 95°C in 0.5°C increments. Two technical replicates of each cDNA sample were analyzed using qPCR. A single no‐reverse‐transcriptase qPCR reaction was performed for each sample in order to detect possible contamination of the RNA with genomic DNA, and a no‐template control was performed, in duplicate, and for every target to check for contaminants. Only technical replicates with a difference of no more than 0.5 cycles were included in the subsequent analysis. Results were only included in the analysis if the difference between the *C*_*q*_ value of the sample and that of the no‐reverse‐transcriptase control or the no‐template control (NTC) was five or larger. *C*_*q*_ values of NTCs were either not defined (as signal did not cross the threshold until cycle 40 or ≥31).

Data analysis was performed according to the ΔΔ*C*_*q*_ method (Pfaffl, 2001) with BioRad CFX Manager software.

### 2.6 Statistical analysis

The fold change values were examined to compare the relative normalized expression of target genes between control and stressor treatments. Statistical analyses were carried out using PAST v3.10 (Hammer, Harper, & Ryan, 2001) software. The fold change values were tested for normal distribution using the Shapiro–Wilk test. Subsequently, the normally distributed data was analyzed using a two‐sample *t*‐test with the level of significance set at *p* ≤ .05. Pearson's *r* and linear regression were used to assess the correlation of Δ*C*_*q*_ values.

## 3 Results

### 3.1 Reference and target genes

Of the six reference genes evaluated, only *GAPDH*,* gltA*,* rpoC1,* and *rpoD* were found to be suitable. The corresponding amplification efficiencies were 101.4%, 97.4%, 93.9%, and 94.8%, respectively (Table S3). *R* ^*2*^ values for the selected reference gene reactions were found to be .996, .998, .998, and .998, respectively (Table S3). Analysis of the melting curves (Fig. S1) and agarose gel electrophoresis (Fig. S2) data demonstrated that the RT‐qPCR reactions were specific. Two of the candidate reference genes tested were disregarded because of a false positive signal (*gyrB*) (data not shown) or low *C*_*q*_ values (*16S rRNA*) (Fig. S4). The selected reference genes (*GAPDH*,* gltA, rpoC1,* and *rpoD*) were included in the reference gene panel for this study, and the suitability of every reference gene for each experimental condition could be confirmed using geNorm (*M* < 0.5).

The two targets (*mcyB* and *mcyD*) were amplified with efficiencies of 97.9% and 93.5% (Table S3), respectively. The *R* ^*2*^ values for the target gene reactions were .998 and .995 (Table S3), respectively. The specificity of the target gene reactions was verified by evaluation of the melting curves (Fig. S1) and agarose gel electrophoresis data (Fig. S2).

### 3.2 Response to elevated temperature

**Figure 1:**
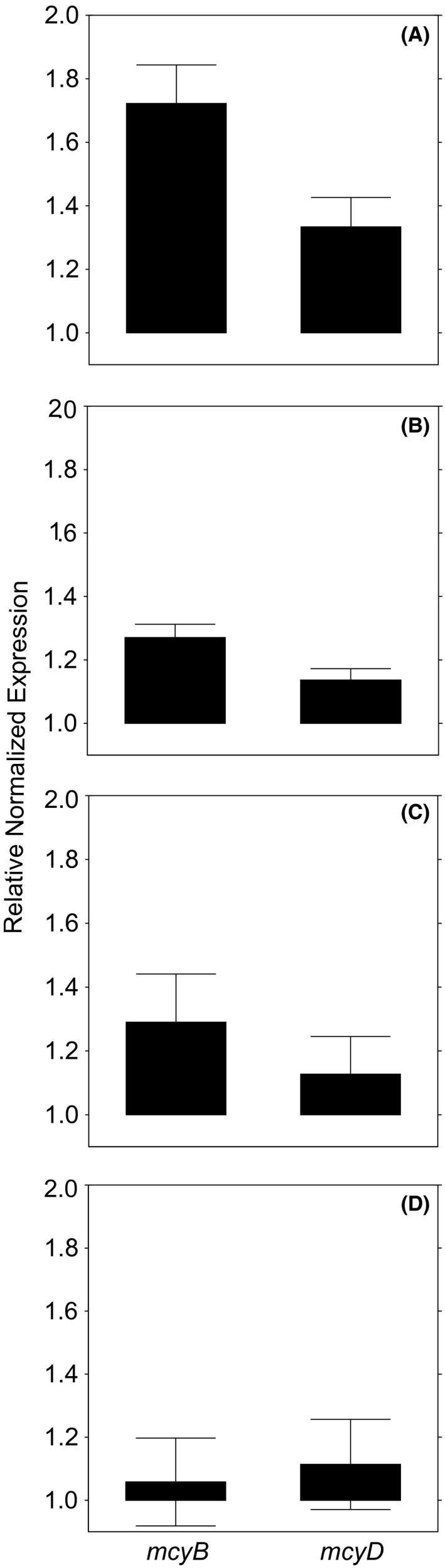
Relative quantification of *mcyB* and *mcyD* gene expression. Error bars indicate standard error of the mean. (A) Elevated temperature. *mcyB* expression (left) was elevated 1.72‐fold when cultures were exposed to 30°C compared with *mcyB* expression in control cultures grown at 20°C. *mcyD* expression (right) was elevated 1.33‐fold when cultures were exposed to 30°C compared with *mcyD* expression in control cultures grown at 20°C. (B) Mixing. *mcyB* expression (left) was elevated 1.27‐fold when cultures were mixed compared with nonmixed cultures. *mcyD* expression (right) was elevated 1.14‐fold when cultures were mixed compared with *mcyB* expression in nonmixed cultures. (C) Low amount of microcystin‐LR. *mcyB* gene expression (left) was elevated 1.29‐fold when 10 μg L^−1^ microcystin‐LR was added to the culture. *mcyD* gene expression (right) was elevated 1.13‐fold when 10 μg l^‐1^ microcystin‐LR was added to the cultures. (D) High amount of microcystin‐LR. *mcyB* gene expression (left) was elevated 1.06‐fold when 60 μg L^−1^ microcystin‐LR was added to the culture. *mcyD* gene expression (right) was elevated 1.11‐fold when 60 μg L^−1^ microcystin‐LR was added to the culture

Elevated temperature was the only stressor tested that induced a significant increase in the expression of one of the microcystin genes, *mcyB*. Relative quantification of the *mcyB* and *mcyD* transcript levels was performed for both temperature conditions. The control condition of 20°C was used as the calibrator (Fig. 1A). For the *mcyB* transcripts, the mean fold change values from control cultures were found to be significantly higher than those of the cultures subjected to elevated temperature (30°C) (mean [standard error of the mean] = 1.00 [.13] vs. 1.72 [.12]; *t* = 3.57, *p* ≤ .05). This indicates that the transcription of the *mcyB* gene was significantly upregulated at the warmer temperature. For the *mcyD* transcripts, however, the mean fold change values from control cultures were not significantly different from those of the cultures subjected to 30°C treatment (1.00 [.15] vs. 1.33 [.09]; *t* = 0.50, *p* = .63). This shows that the expression of *mcyB,* but not that of *mcyD*, was significantly upregulated when cultures were exposed to elevated temperature.

Nonetheless, there was a weak positive correlation between the Δ*C*_*q*_ values of *mcyB* and *mcyD* transcripts (Pearson's *r* = 0.41) under elevated temperature conditions (Fig. S5B). The mean normalized *mcyD* expression level was found to be 77.46% of the mean normalized *mcyB* expression level. The difference between the two was not statistically significant (*t* = 2.10; *p* = .06).

### 3.3 Effect of mixing

Mixing did not significantly alter microcystin gene expression. Relative quantification of *mcyB* and *mcyD* transcript levels was performed for both conditions, mixed and nonmixed. The nonmixed control was used as a calibrator (Fig. 1B). For the *mcyB* transcripts, the mean fold change values from control cultures were not significantly different from those of the cultures subjected to mixing (1.00 [.03] vs. 1.27 [.04]; *t* = 1.72, *p* = .12). Similarly, for the *mcyD* transcripts, the mean fold change values from the control cultures were not significantly different from those of the cultures subjected to stirring (1.00 [.04] vs. 1.14 [0.04]; *t* = 0.92, *p* = .38).

The ΔC_q_ values for the *mcyB* and *mcyD* transcripts under stirring conditions correlated strongly (Pearson's *r* = .97) (Fig. S5C). The mean normalized *mcyD* expression was 89.46% of the mean normalized *mcyB* expression, but the difference was not significant, (*t* = 1.04; *p* = .33).

### 3.4 Effects of the microcystin‐LR concentration

The addition of microcystin‐LR did not significantly alter microcystin gene expression. When investigating the effect of adding 10 μg L^−1^ microcystin‐LR on *mcyB* transcription, we compared the mean fold change values from control cultures with those of the cultures treated with an additional 10 μg L^−1^ microcystin‐LR. The untreated control was used as calibrator (Fig. 1C). The difference in gene expression was not significant (1.00 [.12] vs. 1.29 [.15]; *t* = 1.78; *p* = .09). Similarly, for the *mcyD* transcripts, the mean fold change values of the control and microcystin‐LR‐treated cultures were not significantly different (1.00 [.11] vs. 1.13 [.12]; *t* = 0.51, *p* = .62) (Fig. 1C).

When investigating the effect of adding 60 μg L^−1^ microcystin‐LR on *mcyB* transcription, we compared the mean fold change values from control cultures with those from cultures exposed to 60 μg L^−1^ microcystin‐LR and found that they did not differ significantly (1.00 [.09] vs. 1.06 [0.14]; *t* = 0.24, *p* = .82). The untreated control was used as calibrator (Fig. 1D). The same was true for the *mcyD* transcripts, where the mean fold change values from control cultures were not significantly different than those from cultures exposed to 60 μg L^−1^ microcystin‐LR (1.00 [.08] vs. 1.11 [.14]; *t* = 0.36, *p* = .72) (Fig. 1D).

There was a strong positive correlation between the mean Δ*C*_*q*_ values for *mcyB* and *mcyD* transcripts when low (Pearson's *r* = .76) or high (Pearson's *r* = .97) concentrations of microcystin‐LR were added (Fig. S5 D and E). For the low microcystin concentration, the mean normalized *mcyD* expression was found to be 87.37% of the mean normalized *mcyB* expression, and the difference was not statistically significant (*t* = 0.51; *p* = .62). For the high microcystin concentration, the mean normalized *mcyD* expression was found to be 105.30% of the mean normalized *mcyB* expression, but the difference was not statistically significant (*t* = 0.22; *p* = .83).

## 4 Discussion

Our findings support the hypothesis that elevated temperature leads to increased gene expression of at least one microcystin synthesis gene (*mcyB*). The other hypotheses, presuming a change in microcystin gene expression in response to mixing or additional microcystin, could not be confirmed in this study.

Our results suggest that an increase in microcystin production could possibly be expected with elevated temperatures because we demonstrated that increased temperature led to an upregulation of at least one microcystin synthesis gene. The expression of *mcyB* was significantly upregulated, whereas that of *mcyD* did not change significantly. This shows that a rise in temperature of 10°C does have an effect on the expression of a microcystin synthesis gene (Fig. 1A), increasing our understanding of the answer to the question of how gene expression in *M. aeruginosa* reacts to elevated temperature. Our results, however, are contradictory to some findings that describe a decrease in *mcyB* transcripts by competitive RT‐PCR when comparing 20–30°C (Kim et al., 2005).

Even though some studies suggest otherwise (Helbling et al., 2015), rising global temperatures are thought to favor blooms of toxic algae such as *Microcystis* by affecting several important factors (Paerl & Huisman, 2008, 2009; Paerl & Paul, 2012). First, many potentially toxic cyanobacterial species have a growth advantage at warmer temperatures (Davis et al., 2009; Elliott et al., 2006; Jöhnk et al., 2008). Second, invasive species from tropical regions, such as the toxic cyanobacterium *Cylindrospermopsis raciborskii*, are able to spread to temperate zones (Wiedner, Rücker, Brüggemann, & Nixdorf, 2007). In this study, we now add a third aspect that needs to be considered in the context of rising global temperatures. Because climate change is believed to promote mass occurrences of cyanobacteria, even a small increase in toxin gene expression (Fig. 1A) could amplify the risks posed by toxic cyanobacteria considerably. Even though mixing did not lead to increased microcystin gene expression in this study, additional effects on aquatic ecosystems can be expected due to the effects of climate change on the perturbation patterns of waterbodies. On one hand, increased vertical mixing of the epilimnion caused by more frequent wind stress has been predicted (Gastineau & Soden, 2009; Helbling et al., 2015). On the other hand, model‐based analyses predicted not only an increased water column stability and a warmer epilimnion (Wahl & Peeters, 2014) but also an earlier stratification of waterbodies (Peeters et al., 2007) in response to rising temperatures. Historical trends, furthermore, provide evidence for a shorter annual duration of ice cover of lakes and rivers (Magnuson et al., 2000), a phenomenon connected to earlier onset of phytoplankton blooms (Peeters et al., 2007). Cyanobacteria are well adapted to thrive under those conditions (Huber, Wagner, Gerten, & Adrian, 2012). The elevated cyanobacteria growth will result in higher risks associated with cyanotoxins because elevated temperatures might simultaneously increase the transcription of microcystin genes.

Relevant studies of gene expression in *Microcystis* are often conducted in bioreactors where the cultures are agitated by stirring or a gas mixing system (Makower et al., 2015). This is why learning how mixing affects microcystin gene expression in *M. aeruginosa* is not only interesting from an ecological point of view but also important for assessing the transferability of laboratory‐based results to the field. To the best of our knowledge, this is the first time the effect of mixing on microcystin synthesis gene expression has been explored. Like mixing, the addition of microcystin‐LR could not be shown to impact microcystin gene expression. These findings are in agreement with results from Makower et al. (2015), where the authors found a nonsignificant upregulation of *mcyA* gene when adding 50 μg L^−1^ microcystin‐LR.

The reason why the cell upregulates microcystin synthesis with rising temperature remains unclear. Recent studies suggest that microcystin might have an intracellular function in coping with oxidative stress (Makower et al., 2015). Zilliges et al. (2011) showed that microcystin stabilizes critical proteins such as RubisCO as well as enzymes of the Calvin cycle by binding covalently to their cysteine residues during the oxidative stress conditions caused by high light or H_2_O_2_. Although elevated temperature has not been studied in conjunction with the protective function of microcystin, it is easy to envision that the protein‐modulating function of microcystin is beneficial at higher temperatures and in conjunction with high light conditions, like in this study. Such a mechanism could also explain the selective advantage of toxic *Microcystis* genotypes over nontoxic ones at elevated temperatures (Davis et al., 2009).

In this study, we introduced a novel RT‐qPCR assay for the relative quantification of microcystin gene expression in *M. aeruginosa*. We established a new reference gene panel consisting of four reference genes: *GAPDH*,* gltA*,* rpoC1,* and *rpoD*. To the best of our knowledge, this is the first time this unique combination of reference genes was used to investigate gene expression in *Microcystis*. A reference gene panel should be preferred over a single reference gene because it is more resilient to small fluctuations in expression stability. Ideally, reference genes should have a range of expression similar to that of the target genes. Therefore, the 16S rRNA gene, which was also considered as a reference gene in this study, was found not to be suitable due to its high expression levels (Fig. S4). This is in agreement with other studies that assessed this gene as a candidate reference gene (Tran et al., 2013). In addition, extremely low *C*_*q*_ values lead to technical difficulties with baseline correction of the amplification curves (data not shown). On the other hand, using the 16S rRNA gene as reference gene worked for others (Kuniyoshi et al., 2013; Ngwa et al., 2014; Pimentel & Giani, 2014; Sevilla et al., 2008). It is good practice to assess the suitability of a reference gene for each new experimental condition or treatment. All four reference genes presented here were found to be suitable reference genes, that is, they were stably expressed under the conditions tested in this study. Therefore, the reference gene panel presented here is a valuable starting point for finding suitable reference genes for future experimental studies. By offering four reference genes to choose from, there is an increased chance of identifying at least one stable reference gene for other experimental conditions.

Two different target genes, *mcyB* and *mcyD,* were used in this assay. Both are suitable target genes for several reasons. First, they are both essential for the synthesis of microcystin (Dittmann, Neilan, Erhard, von Döhren, & Börner, 1997; Nishizawa et al., 2000; Tillett et al., 2000). Second, because they are located on two bidirectionally transcribed operons of the microcystin gene cluster, they represent both these operons (Tillett et al., 2000). Finally, primer sequences for these genes are available from previous studies (Kurmayer & Kutzenberger, 2003; Sevilla et al., 2008). Although the primers for *mcyD* had been used for gene expression studies before (Kuniyoshi et al., 2013; Pimentel & Giani, 2014; Sevilla et al., 2008, 2010), those for *mcyB* had only been used for absolute quantification in qPCR studies (Kurmayer & Kutzenberger, 2003). To the best of our knowledge, the qPCR assay introduced here is the first to target both of these genes at the same time. To measure two target genes from the microcystin gene cluster gives an idea of the risks associated with certain environmental stressors, potentially even before microcystin is produced. Although most studies target only one gene from the microcystin gene cluster, the two target genes we choose here represent not only the two operons of the microcystin gene cluster but also target representative nonribosomal peptide synthase (*mcyB*) and polyketide synthase (*mcyD*) genes.

Both genes were found to be suitable target genes, and their expression correlated well in most experiments (see Fig. S5). This means that they are largely coexpressed. The coexpression of *mcyB* and *mcyD* is explained by the fact that both genes encode proteins of a common multienzyme complex that performs the nonribosomal synthesis of microcystin. It is notable that the relative normalized expressions of *mcyB* and *mcyD* differed consistently in our experiments. This is an interesting observation even though the differences were not significant, drawing attention to the fact that the mechanisms regulating microcystin production are still largely unknown. The target genes are on different polycistronically transcribed operons and are, therefore, potentially subjected to differential regulation. The microcystin synthesis genes may be regulated by light‐dependent alternate transcription start points like those found upstream of *mcyA* and *mcyD*, or their regulation may be governed by intercistronic promoters (Kaebernick, Dittmann, Börner, & Neilan, 2002). In addition, putative binding boxes for the ferric uptake regulator Fur (Martin‐Luna et al., 2006) and the global nitrogen regulator NtcA (Ginn, Pearson, & Neilan, 2010) in the microcystin gene cluster suggest transcriptional regulation in response to nutrients. These regulatory mechanisms imply regulation of the microcystin genes on the transcript level, emphasizing the relevance of transcriptional studies.

Although no microcystin measurements were performed in this study, it has been shown that expression of microcystin synthesis genes correlates with levels of microcystin in some studies (Kuniyoshi et al., 2013; Pimentel & Giani, 2014; Sevilla et al., 2008), while other studies could not show this connection (Kaebernick et al., 2000; Ngwa et al., 2014; Sipari, Rantala‐Ylinen, Jokela, Oksanen, & Sivonen, 2010; Wood et al., 2011). Microcystin measurements can also be misleading because the lag phase between transcription and actual microcystin formation remains unknown and because protein‐bound microcystin might be missed (Zilliges et al., 2011). Although other research on the effects of elevated temperature have mostly concentrated on the presence of potentially toxigenic cyanobacteria (Davis et al., 2009; Dziallas & Grossart, 2011) or the amount of microcystin itself (Dziallas & Grossart, 2011; Kleinteich et al., 2012; Sivonen, 1990; van der Westhuizen & Eloff, 1985), our research bridges the gap between the two by focusing on gene expression.

## 5 Conclusion

For the first time, an RT‐qPCR assay was used to explore three environmental factors connected to climate change. The results revealed that increased temperature leads to the upregulation of the microcystin synthesis gene *mcyB*, whereas mixing and the addition of microcystin‐LR does not cause increased expression of *mcyB* or *mcyD*. This led us to conclude that future rising temperatures may increase the risks associated with toxic cyanobacteria by influencing microcystin gene expression, whereas changed perturbation patterns and increased microcystin levels might not increase the risks. To make even more substantiated predictions about the effect of climate change on microcystin synthesis gene expression, other factors connected to and caused by global warming need to be explored in future studies.

## Funding Information

This work was supported by the German Research Foundation (DFG) through the TUM International Graduate School of Science and Engineering (IGSSE). This work was also supported by the German Research Foundation (DFG) and the Technische Universität München within the funding programme Open Access Publishing.

## Conflict of Interest

None declared.
