# Supplementary material for: Influence of temperature, mixing, and addition of microcystin‐LR on microcystin gene expression in Microcystis aeruginosa
Source: Microbiologyopen. 2016 Jul 14;6(1):e00393. doi: 10.1002/mbo3.393 (PMC5300888; doi:10.1002/mbo3.393)

Supplemental Information

Table S1. Quantification, purity and yield of RNA

Sample	Experiment	RNA concentration (ng/µl)	RNA yield (ng)	A260/280
C1	Elevated temperature	710.0	35500	1.955
C2		738.4	36920	1.960
C3		498.8	24940	1.986
C4		490.0	24500	1.917
C5		293.6	14680	1.756
C6		199.6	9980	1.658
T1		598.0	29900	1.952
T2		628.0	31400	1.967
T3		454.4	22720	1.929
T4		460.0	23000	1.904
T5		211.6	10580	1.734
T6		400.0	20000	1.835
C1	Mixing	198.8	9940	1.821
C2		134.8	6740	1.728
C3		110.4	5520	1.663
C4		348.4	17420	1.914
C5		98.4	4920	1.577
C6		248.0	12400	1.787
T2		182.8	9140	1.785
T3		242.8	12140	1.851
T4		494.0	24700	1.963
T5		434.0	21700	1.874
T6		407.2	20360	1.924
C1	Low amount of microcystin-LR	415.2	20760	2.043
C2		364.8	18240	2.018
C3		438.8	21940	2.024
C4		490.0	24500	1.911
C5		390.0	19500	1.912
C6		495.6	24780	1.945
C7		376.0	18800	1.843
C8		502.4	25120	1.953
C9		491.2	24560	1.949
T1		404.0	20200	2.004
T2		310.4	15520	2.047
T3		398.0	19900	2.026
T4		598.0	29900	1.944
T5		386.0	19300	1.885
T6		211.6	10580	1.706
T7		606.4	30320	1.961
T8		351.6	17580	1.923

T9		278.0	13900	1.878
C1	High amount of microcystin-LR	241.2	12060	1.822
C2		274.0	13700	1.856
C3		229.2	11460	1.808
C4		490.0	24500	1.911
C5		390.0	19500	1.912
C6		495.6	24780	1.945
C7		376.0	18800	1.843
C8		502.4	25120	1.953
C9		491.2	24560	1.949
T1		188.0	9400	1.787
T2		260.8	13040	1.842
T3		230.4	11520	1.852
T4		466.8	23340	1.891
T5		480.0	24000	1.846
T6		101.6	5080	1.649
T7		332.8	16640	1.93
T8		488.8	24440	1.974
T9		275.6	13780	1.847

Table S2. Criteria for reference gene primer pair

Tested parameter	Pass Criteria
Specificity *in silico*	BLAST search does not identify an alternate biding site in cyanobacterial genome
Specificity *in vitro*	Single band of expected size on agarose gel<br>Melting curve examination /single melting peak<br>No false positive results
Similar product quantity as target genes	C_q values similar to C_q of target genes<br>Baseline must be present
Annealing temperature	Can be used at a temperature as the target genes
Stable expression	M values < 0.5 (geNorm)
Efficiency of reaction	Between 90 and 110%
R^2 value of reaction	> 0.980

Table S3. Efficiency and R^2 values of qPCR reactions and amplicon size for target and reference genes

Primer name	Target gene	Efficiency (%)	R^2	Amplicon length (bp)
**mcyB30F**	*mcyB* peptide synthetase	97.9	0.998	97
**mcyB108R**				
**RmcyDF**	*mcyD* polyketide synthase	93.5	0.995	80
**RmcyDR**				
**GAPDH727F**	*GAPDH* glyceraldehyde-3-phosphate dehydrogenase	101.4	0.996	99
**GAPDH825R**				
**gltA429F**	*gltA* citrate synthase	97.4	0.998	100
**gltA528R**				
**rpoC1F**	*rpoC1* DNA-directed RNA polymerase subunit gamma	93.9	0.998	181
**rpoC1R**				
**rpoD230F**	*rpoD* RNA polymerase sigma factor RpoD	94.8	0.998	125
**rpoD354R**				

Figure S1. Melting curves profiles of target and reference gene PCR products. Genes: (A) *mcyB*, (B) *mcyD*, (C) *GAPDH*, (D) *gltA*, (E) *rpoC1*, and (F) *rpoD*.

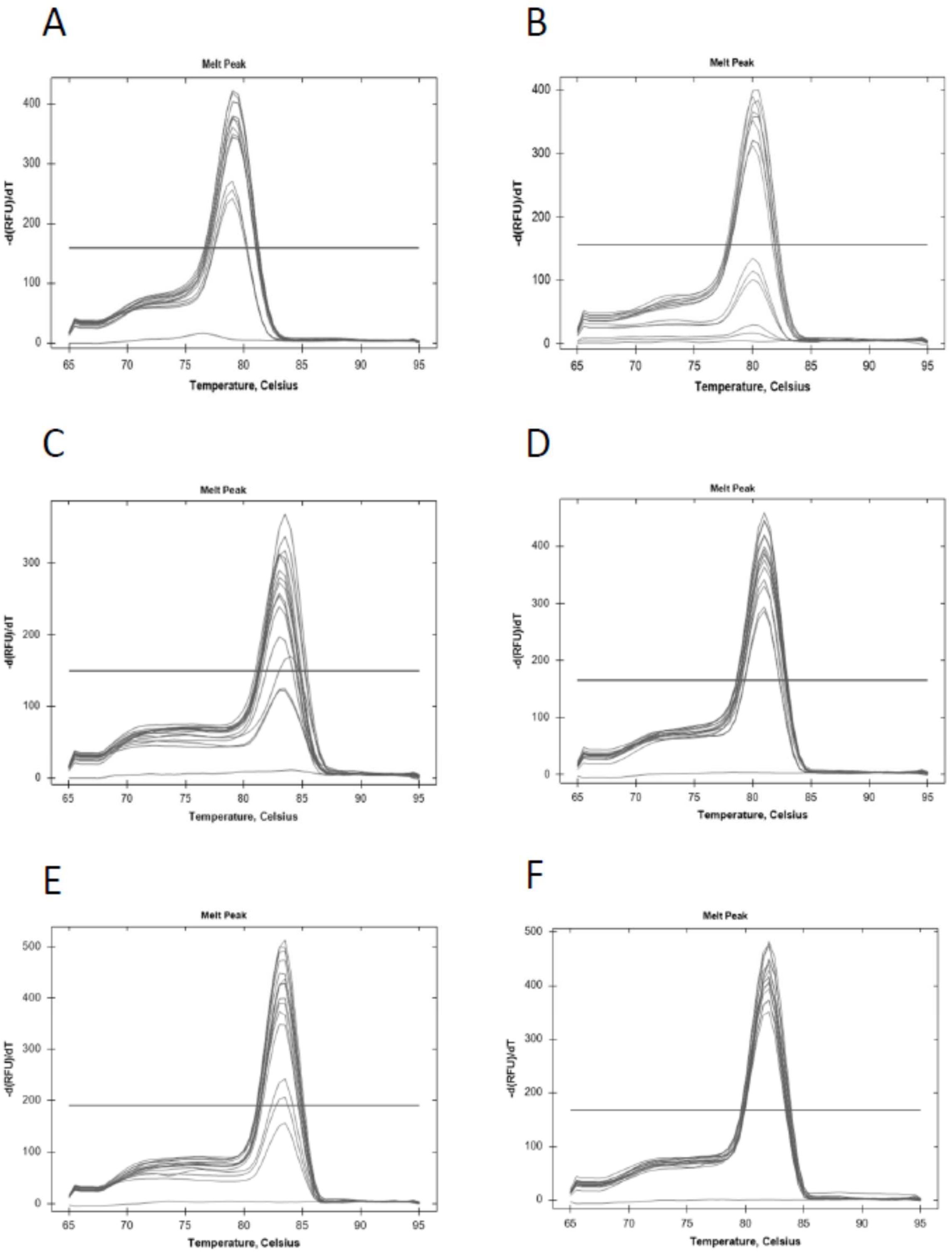

Figure S2. qPCR products on 3% agarose gel. (1) GeneRuler DNA Ladder Mix. (2) *mcyB* qPCR product. (3) *mcyD* qPCR product. (4) *GAPDH* qPCR product. (5) *gltA* qPCR product. (6) *rpoC1* qPCR product. (7) *rpoD* qPCR product. (8) no-template control. (9) GeneRuler DNA Ladder Mix.

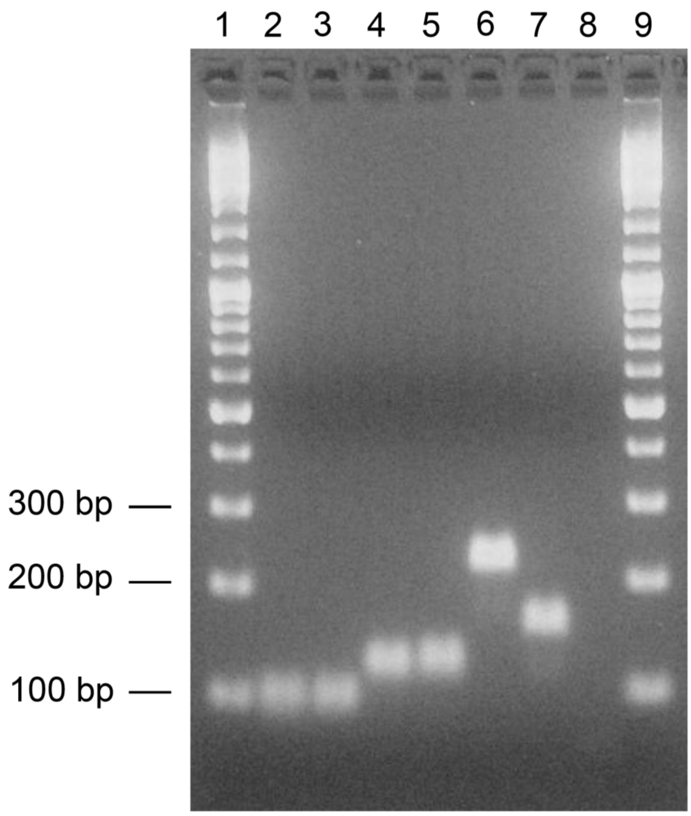

Figure S3. Standard curves of target and reference gene PCR products. Genes: (A) *mcyB*, (B) *mcyD*, (C) *GAPDH*, (D) *gltA*, (E) *rpoC1*, and (F) *rpoD*.

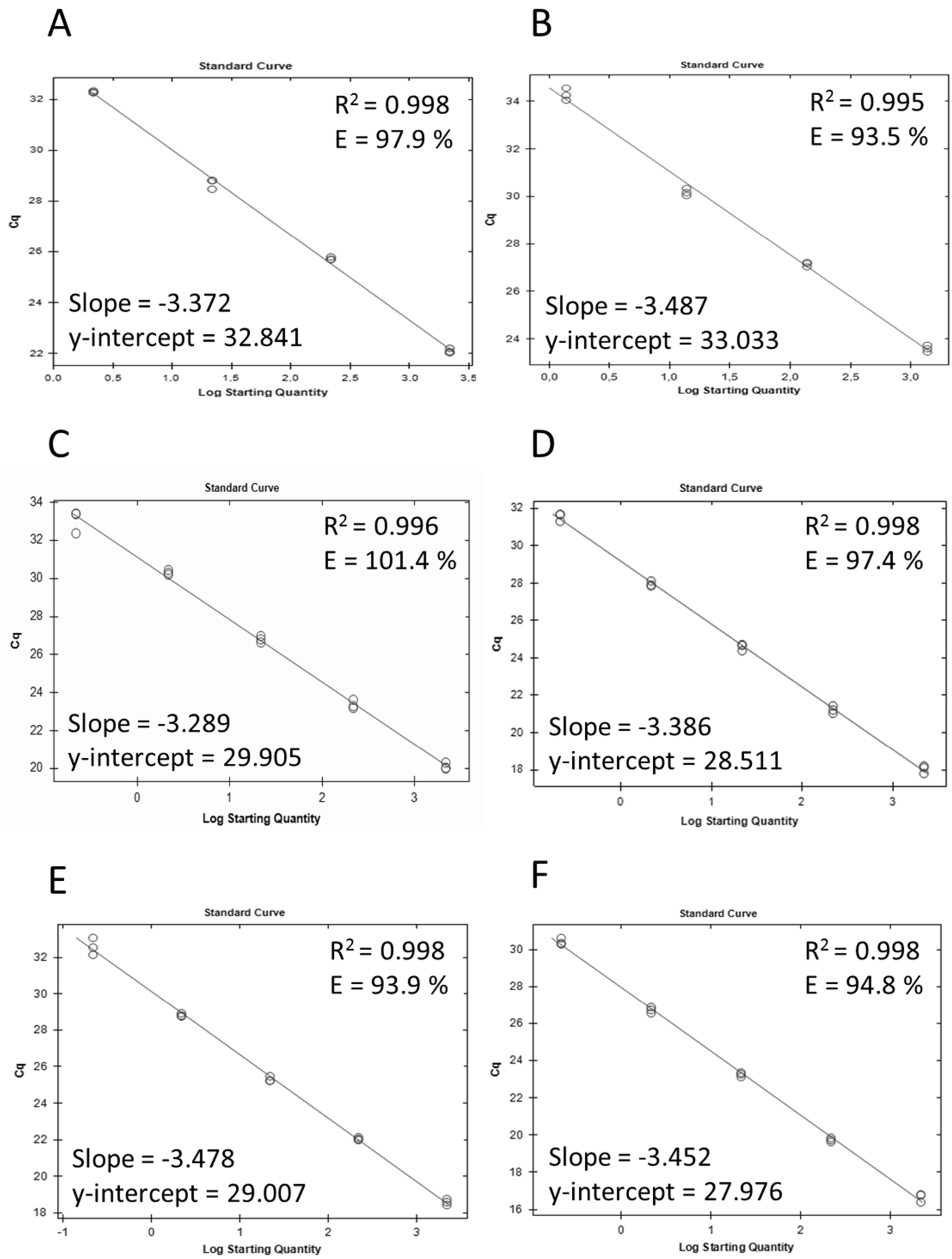

Figure S4. C_q values of reference and target gene candidates (n=4) obtained during assay optimisation.

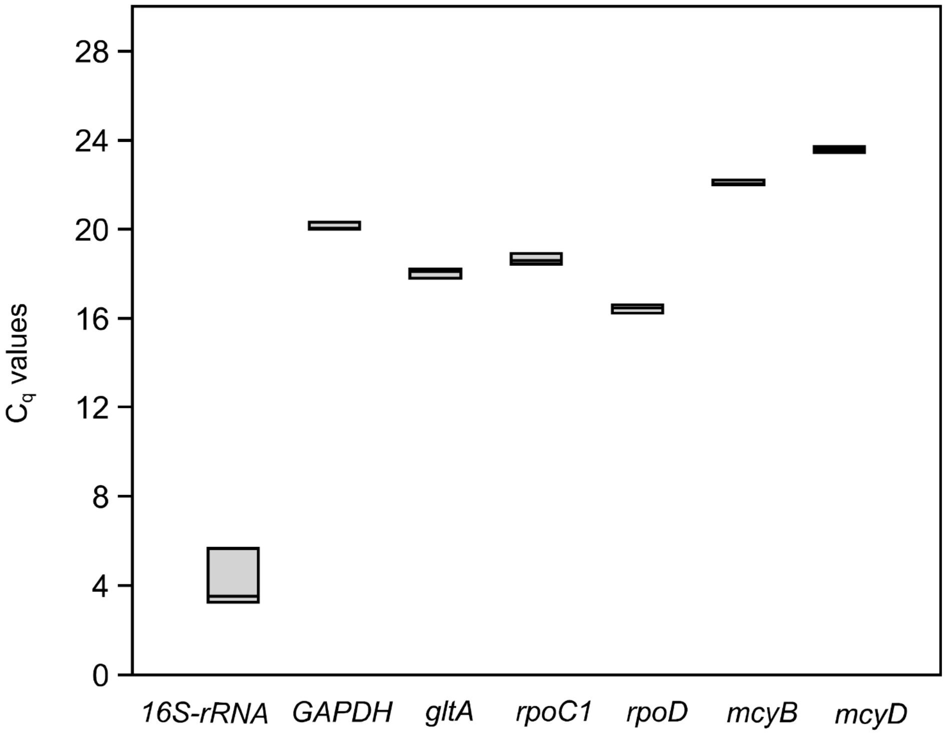

Figure S5. Correlation analysis for ΔC_q values of *mcyB* and *mcyD* transcripts. (A) Strong positive correlation of *mcyB* and *mcyD* ΔC_q values for all tested experimental conditions (Pearson's r = 0.75). (B) Weak positive correlation of *mcyB* and *mcyD* ΔC_q values for temperature experiment (Pearson's r = 0.41). (C) Strong positive correlation of *mcyB* and *mcyD* ΔC_q values for the mixing experiment (Pearson's r = 0.97). (D) Strong positive correlation of *mcyB* and *mcyD* ΔC_q values for the microcystin-LR 10 µg/l experiment (Pearson's r = 0.76). (E) Strong positive correlation of *mcyB* and *mcyD* ΔC_q values for the microcystin-LR 60µg/l experiment (Pearson's r = 0.97).

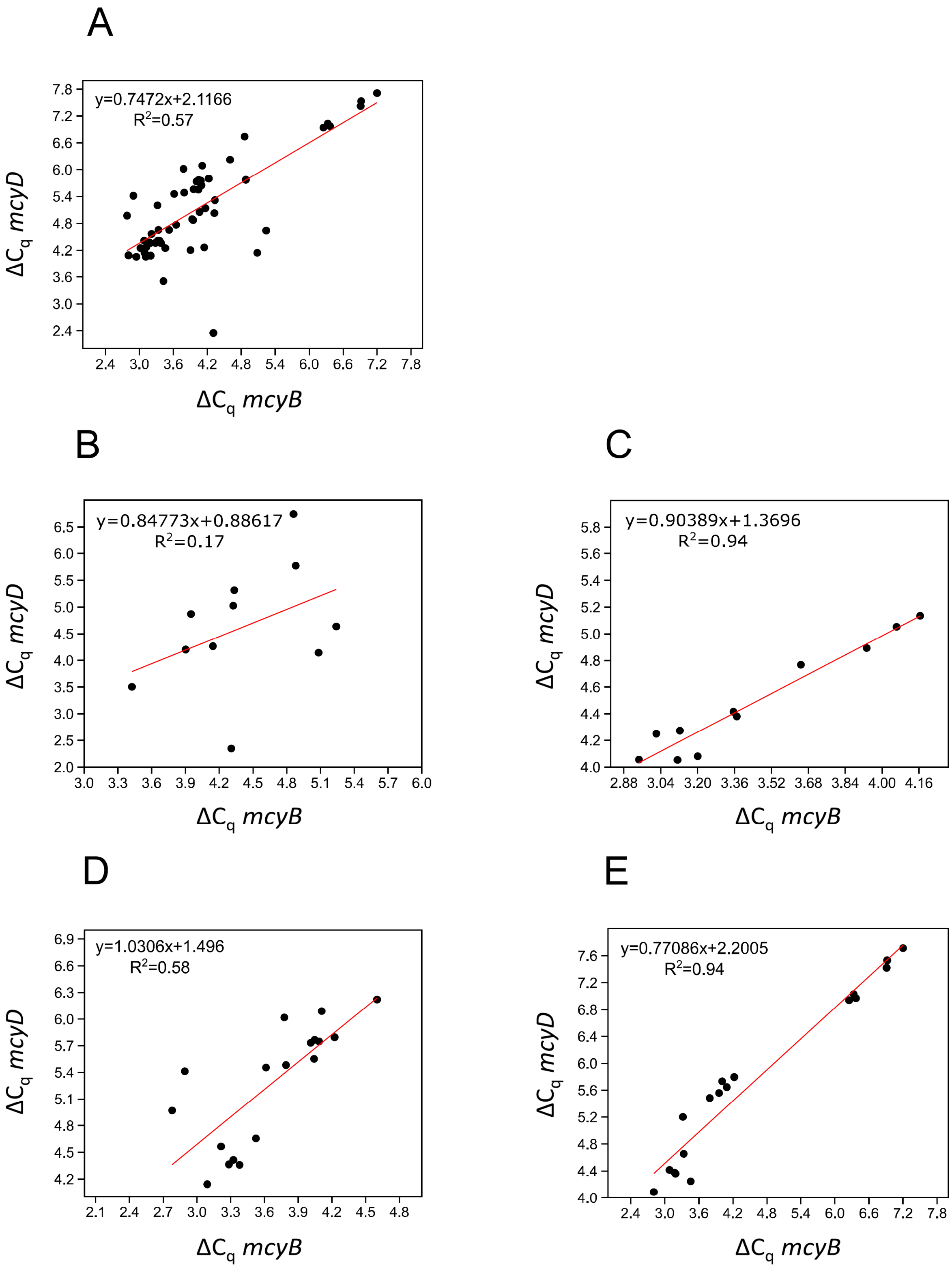

Supplement: Supplementary file 1 [file MBO3-6-0-s001.pdf]
